# Refractory Diarrhea Related to EPHB4 Mutation in a Patient With Capillary Malformation-Arteriovenous Type 2 Syndrome

**DOI:** 10.14309/crj.0000000000001707

**Published:** 2025-06-04

**Authors:** Bridgette B. McNally, Margaret C. Liu, Tisha Lunsford, Jennifer Horsley-Silva, Karen Swanson, Thomas Byrne, Marcela Salomao, Maxwell Smith, Rosita Frazier

**Affiliations:** 1 Department of Gastroenterology, Mayo Clinic Arizona, Phoenix, AZ; 2 Department of Pulmonology, Mayo Clinic Arizona, Phoenix, AZ; 3 Department of Hepatology, Mayo Clinic Arizona, Phoenix, AZ; 4 Department of Pathology, Mayo Clinic Arizona, Phoenix, AZ

**Keywords:** malabsorption, failure to thrive, capillary malformation-arteriovenous type 2 syndrome

## Abstract

Capillary malformation-arteriovenous type 2 syndrome (CM-AVM2) is a rare, genetic vascular disorder associated with abnormal capillary malformations and arteriovenous malformations. We present a case of CM-AVM2 with refractory diarrhea and failure to thrive due to aberrant, telangiectatic capillaries, and subepithelial collagen deposition found throughout the GI tract on bidirectional endoscopy. The patient was recalcitrant to standard therapies, so bevacizumab was trialed, but the patient expired. This is the first case of CM-AVM2 with diffuse GI mucosal involvement resulting in malabsorption. There is no treatment for this pathology, but treatment with bevacizumab or a mammalian target of rapamycin inhibitor may be efficacious.

## INTRODUCTION

Capillary malformation-arteriovenous (CM-AVM) is an autosomal dominant, congenital condition that results in the formation of arteriovenous malformations (AVMs) in the extremities, face, brain, and spinal cord.^1,2^ About 50%–68% of patients with CM-AVM have a loss-of-function variant of *RASA1*, which results in dysregulation of angiogenesis by interfering with regulation of mitogen-activated protein kinase. Pathogenic variants of *EPHB4* define CM-AVM type 2 syndrome (CM-AVM2). *EPHB4* is a transmembrane tyrosine kinase receptor that promotes vasculogenesis, proper morphogenesis of capillary beds, and embryologic neuronal development. Inactivation of *EPHB4* results in the upregulation of mammalian target of rapamycin (mTOR), which leads to vascular overgrowth and the development of AVMs and CMs.^1–3^ In CM-AVM2, capillary malformations (CMs) are classically present on the trunk and extremities and also found in the bone, central nervous system, and liver. The presence of oral telangiectasias was previously noted in 12% of patients with CM-AVM2, and a previous study also demonstrated the presence of recurrent epistaxis and telangiectasias in the setting of classic CMs consistent with CM-AVM2.

Hereditary hemorrhagic telangiectasia (HHT) is an autosomal dominant vascular malformation disorder characterized by telangiectasias of the oral cavity, lips, nose, and gastrointestinal tract with AVMs in the lung, liver, and brain.^2^ Despite the similar manifestations in CM-AVM2 and HHT, they have distinct pathophysiology and clinical manifestations. Notably, on genetic sequencing of patients presenting with classic features of HHT without the classic gene variants associated with HHT, the presence of a pathologic *EPHB4* gene variant was identified and led to subsequent diagnosis of CM-AVM2 syndrome. Notably, there is no approved treatment of CM-AVM2 syndrome.

We present a unique case of a 20-year-old man with CM-AVM2 syndrome who was found to have diffuse, microscopic gastrointestinal involvement resulting in refractory diarrhea with malabsorption and failure to thrive (FFT).

## CASE REPORT

A 20-year-old man with CM-AVM2 with hepatic involvement and FTT presented to a tertiary referral center for evaluation of subacute diarrhea.

Three months prior, the patient noted frequent, nonspecific diarrhea that became bloody, and he was later admitted for hemorrhagic shock due to a variceal hemorrhage. At that time, abdominal imaging identified innumerable hepatic hemangiomas and AVMs, concerning for a vascular anomaly, prompting genetic testing. Whole-genome sequencing identified a pathologic variant of the *EPHB4* (c.128_134dup (p.Ser46Glyfs*7)), solidifying the diagnosis of CM-AVM2. The patient was adopted in infancy, so family history of a pathologic variance in *EPHB4* was unknown.

However, the patient had persistent diarrhea and a 16 kg weight loss, so he was admitted to an outside hospital. Stool testing was consistent with secretory diarrhea, and a colonoscopy showed lymphocytic aggregates and subepithelial collagenous deposits isolated to a biopsy of a cecal polyp, but no intraepithelial lymphocytosis or collagen bands were identified; there was no evidence of amyloid. Therefore, the patient was placed on budesonide for concerns of collagenous colitis. Despite steroids, antidiarrheals and bowel rest with total parenteral nutrition, his diarrhea, weight loss, electrolyte derangements, hypophosphatemia, and hypoalbuminemia persisted, prompting transfer to our center.

**Figure 1.**
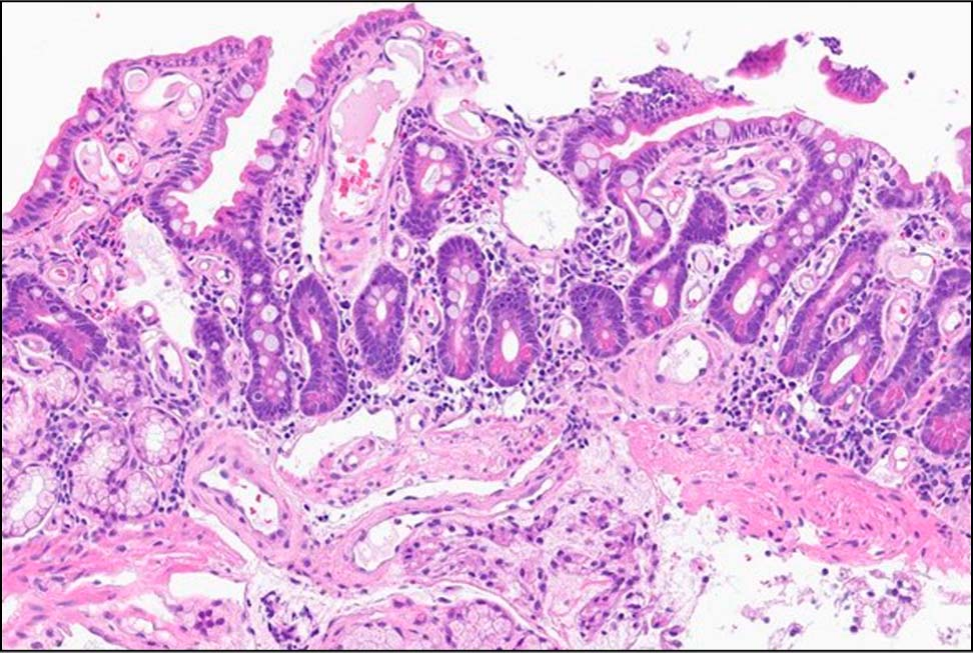
Duodenal biopsy. Diffuse, aberrant, and telangiectatic lamina propria capillaries in the background of mild villous atrophy (200×).

**Figure 2.**
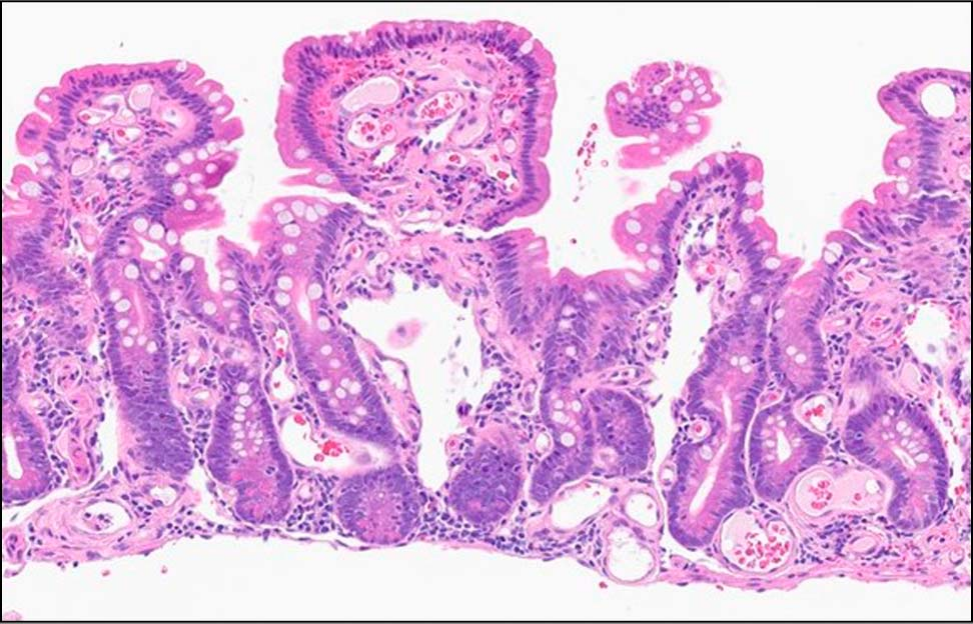
Jejunal biopsy. Diffuse, aberrant, and telangiectatic lamina propria capillaries in the background of mild villous atrophy (200×).

**Figure 3.**
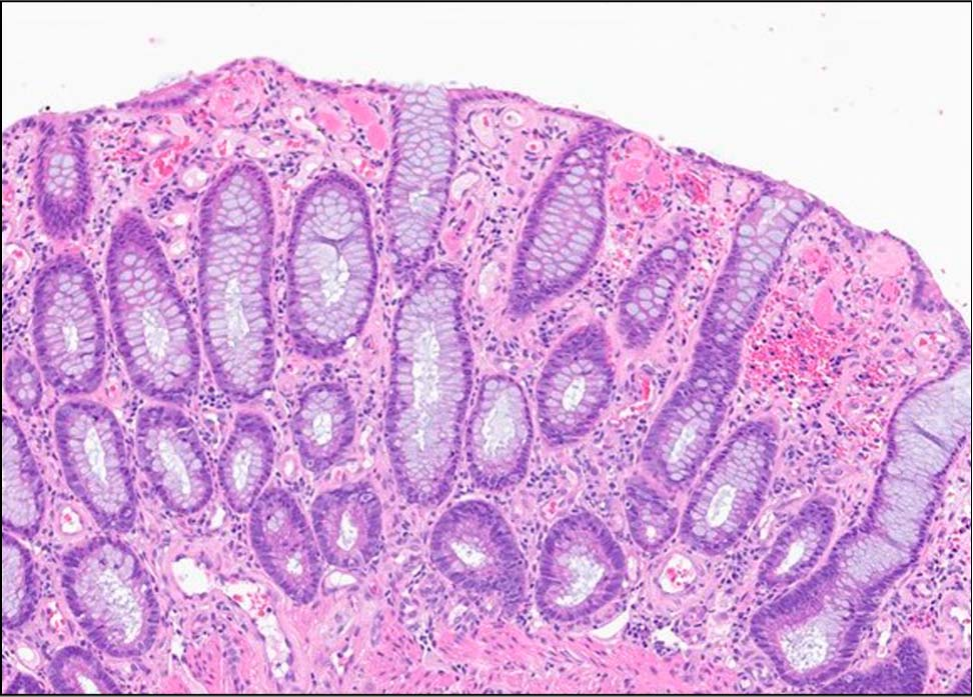
Ascending colon biopsy. Diffuse, aberrant, and telangiectatic lamina propria capillaries (160×).

On arrival, the patient's body mass index was 12. Push enteroscopy and colonoscopy demonstrated small esophageal varices, white nummular gastric lesions, scattered mucosal ulcerations, and erythematous mucosa at the ileocecal valve, but there was no endoscopic evidence of AVMs or aberrant vasculature. Histology demonstrated diffuse involvement of aberrant and telangiectatic capillaries throughout the entire GI tract, with villous atrophy in the small bowel, suggestive of a multisystem vascular anomaly (Figures 1–3). Given that the biopsies did not demonstrate collagenous colitis, the patient was weaned off steroids. Other etiologies of secretory diarrhea such as infection, carcinoid, neuroendocrine tumor, pancreatic insufficiency, protein losing enteropathy, and Whipple's disease were ruled out. Given persistent diarrhea with FTT, recalcitrant to steroids, antidiarrheals, and total parenteral nutrition, a multidisciplinary discussion led to compassionate use of bevacizumab to target the underlying vascular process. Unfortunately, the patient passed away after only 2 doses.

## DISCUSSION

We report the first case of CM-AVM2 with diffuse GI mucosal involvement with abnormally formed capillaries, dilated/telangiectatic lesions leading to small bowel villous atrophy and subepithelial collagen deposition, causing large volume secretory diarrhea due to decreased intestinal surface area and absorptive capacity.

It has been previously shown that *EPHB4* is essential for the development of lymphatic vessels, and pathologic variants in *EPHB4* can result in defective lymphatic venous valves and generalized lymphatic dysplasia.^4^ It has been primarily shown that this results in lymphedema, including hydrops fetalis, pleural effusions, and complications in placental development. While our case did not show aberrant lymphatic vessels in the small intestine, this should be considered and ruled out in patients who present with FTT and known pathologic variants of *EPHB4* given the association between *EPHB4* and lymphatic vessels.

Bevacizumab is a vascular endothelial growth factor inhibitor used in cases of HHT with clinically significant bleeding and/or high output heart failure. While our patient did not have a diagnosis of HHT, nor did he have the presence of high output heart failure or gastrointestinal hemorrhage, bevacizumab was compassionately used with the goal of inhibiting further development of aberrant telangiectasias and capillaries in the small intestine, preventing further malabsorption. However, this patient ultimately died from complications of FTT despite 2 doses of bevacizumab.

Given that the pathophysiology CM-AVM2 results from a pathologic variant of the *EPHB4* gene, causing upregulation of mTOR and subsequent vascular overgrowth and the development of AVMs and CMs, investigation of the use of systemic mTOR inhibitors (sirolimus, everolimus, and temsirolimus) in patients with *EPHB4* variants warrants investigation to further identify the phenotypes and clinical presentations of patients with pathologic variants of *EPHB4* in CM-AVM2 syndrome.

## DISCLOSURES

Author contributions: BB McNally manuscript writing, MC Liu manuscript editing, T. Lunsofrd manuscript editing, J. Horsley-Silva manuscript editing, M. Salomao provided images, M. Smith provided images, R. Frazier—manuscript editing and is the article guarantor.

Financial disclosure: None to report.

Informed consent was obtained for this case report.

Previous presentation: This case was presented at American College of Gastroenterology Annual Scientific Meeting; Philadelphia, PA; October 27, 2024; Presidential Poster Award Recipient.

## References

[1] Amyere M Revencu N Helaers R . Germline loss-of-function mutations in EPHB4 cause a second form of capillary malformation-arteriovenous malformation (CM-AVM2) deregulating RAS-MAPK signaling. Circulation. 2017;136(11):1037–48. 28687708 10.1161/CIRCULATIONAHA.116.026886

[2] Yu J Streicher JL Medne L Krantz ID Yan AC. *EPHB4* mutation implicated in capillary malformation-arteriovenous malformation syndrome: A case report. Pediatr Dermatol. 2017;34(5):e227–30. 28730721 10.1111/pde.13208

[3] Wooderchak-Donahue WL Akay G Whitehead K . Phenotype of CM-AVM2 caused by variants in EPHB4: How much overlap with hereditary hemorrhagic telangiectasia (HHT)?. Genet Med. 2019;21(9):2007–14. 30760892 10.1038/s41436-019-0443-z

[4] Martin-Almedina S Martinez-Corral I Holdhus R . EPHB4 kinase-inactivating mutations cause autosomal dominant lymphatic-related hydrops fetalis. J Clin Invest. 2016;126(8):3080–8. 27400125 10.1172/JCI85794 PMC4966301

